# The dynamics of physical and mental health in the older population

**DOI:** 10.1016/j.jeoa.2016.07.002

**Published:** 2017-06

**Authors:** Julius Ohrnberger, Eleonora Fichera, Matt Sutton

**Affiliations:** Manchester Centre for Health Economics, Institute of Population Health, Jean McFarlane Building, University of Manchester, Oxford Road, Manchester M13 9PL, UK

**Keywords:** Mental health, Physical health, Dynamic models, Cross-effects

## Abstract

Mental and physical aspects are both integral to health but little is known about the dynamic relationship between them. We consider the dynamic relationship between mental and physical health using a sample of 11,203 individuals in six waves (2002–2013) of the English Longitudinal Study of Ageing (ELSA). We estimate conditional linear and non-linear random-effects regression models to identify the effects of past physical health, measured by Activities of Daily Living (ADL), and past mental health, measured by the Centre for Epidemiological Studies Depression (CES-D) scale, on both present physical and mental health. We find that both mental and physical health are moderately state-dependent. Better past mental health increases present physical health significantly. Better past physical health has a larger effect on present mental health. Past mental health has stronger effects on present physical health than physical activity or education. It explains 2.0% of the unobserved heterogeneity in physical health. Past physical health has stronger effects on present mental health than health investments, income or education. It explains 0.4% of the unobserved heterogeneity in mental health. These cross-effects suggest that health policies aimed at specific aspects of health should consider potential spill-over effects.

## Introduction

Despite the fact that both mental and physical aspects are considered by the World Health Organisation (WHO) as integral dimensions of health and well-being (WHO, 2013a) little is known about the dynamic relationships between them. Examining this relationship could have important implications for understanding more about the determinants of either health dimension and for evaluating the indirect effects of mental health policies on physical health and physical health policies on mental health.

A large literature considers socio-economic factors, lifestyle behaviours and social network, amongst others, as important determinants of health. A number of studies find a positive association of socio-economic factors such as income and employment on health, the so-called “income-health” gradient (Van Doorslaer and Koolman, 2004, Llena-Nozal et al., 2004, Stuckler et al., 2009, Allen et al., 2014). Some studies find that retirement has a negative association with both mental and physical health (Dave et al., 2006). Education has been found to have a positive association with physical and mental health (the so-called “education-health” gradient) because individuals produce their health more efficiently and allocate their inputs of production better (Cutler and Lleras-Muney, 2006).

Heath behaviours also contribute in determining physical and mental health (Cutler and Lleras-Muney, 2006), with physical activity showing positive correlation with both better physical health (Jeoung et al., 2013, Durstine et al., 2013, Contoyannis and Jones, 2004) and lower depressive symptoms (Galper et al., 2006). Social networks are positively associated with better mental health outcomes, especially for older people (Allen et al., 2014) and show a negative association with the risk of mortality (Holt-Lunstad et al., 2010).

However, little is known about the dynamic cross-effects between mental health and physical health. Mental health could be an important determinant of physical health because individuals with depressive symptoms, for example, might be less efficient producers of their physical health. There may also be negative spillovers of poor physical health on mental health.

Several studies have investigated the dynamics of health (see for example Contoyannis et al., 2004, Jones et al., 2006, Contoyannis and Li, 2011, Allin and Stabile, 2012). Contoyannis et al. (2004) examined the determinants of self-assessed health (SAH) in eight waves of the British Household Panel Survey with a dynamic model. They found self-assessed health to be characterised by substantial positive state dependence and that unobservable heterogeneity accounted for approximately 30 percent of the unexplained variation in SAH.

Only two studies have examined the dynamics of mental health explicitly (Roy and Schurer, 2013, Hauck and Rice, 2004). Hauck and Rice (2004), investigated the determinants of mental health, measured by the General Health Questionnaire (GHQ) score, in eleven waves of the British Household Panel Survey. They found positive state dependence in mental health, with unobservable heterogeneity accounting for approximately 20 percent of the total unexplained variation in mental health.

Some cross-sectional studies have found mental health to be a strong correlate of physical health (see for example Rowan et al., 2005, Nabi et al., 2008, Surtees et al., 2008), but there are no studies that examine the dynamic relation between the two. Our aim is to investigate the relation between mental and physical health while accounting for the dynamics of physical health and mental health. Modelling the interrelation between mental and physical health could reduce the unexplained variation in health found by Contoyannis et al. (2004). It can also have important policy implications for the indirect effects of mental and physical health interventions. Significant cross-effects would imply that health policies should consider these spill-overs in health interventions. Strong state dependence in either health dimension would suggest that short run health interventions have strong persistence over time, because poor past health may have effects later in life. On the contrary, low state dependence in health would suggest that health interventions have one-off effects, indicating higher variability in health over time.

In a Grossman-type model (1972) one could think of there being two health capital stocks, one each for mental and physical health. Each of these stocks will be interdependent such that health (either physical or mental) depends on both physical and mental health and other health investment activities. We estimate a reduced form of this equation using conditional linear and non-linear dynamic models in six waves of the English Longitudinal Study of Ageing. As a measure of physical health, we use the Activities of Daily Living (ADL), a six-item scale variable assessing whether an individual can perform tasks of daily living or not. As a measure of mental health, we use an eight-item version of the Centre for Epidemiological Studies Depression (CES-D) Scale.

To preface our results we find that both outcome variables are moderately state dependent. Both mental and physical health are responsive to their own past stocks. We find significant positive cross effects of physical with mental health. Including past physical health in the mental health equation shows stronger effects than, for instance, income or health investments. Unobserved heterogeneity in mental health is reduced by 0.4%. Past mental health shows a stronger effect on present physical health than deterministic health variables such as education and physical activity. Unobserved heterogeneity is reduced by 2%.

## Data and summary statistics

### The English Longitudinal Study of Ageing (ELSA)

We use six waves of data from the ELSA (2002–2013). The ELSA is an ongoing longitudinal survey of a representative sample of English population aged 50 years and older, and was designed to collect objective and subjective data relating to health, socio-economic circumstances, and well-being once every two years. The sample was drawn from households that participated in the Health Survey for England (HSE) and agreed to be followed-up. The first wave was conducted between March 2002 and March 2003 and consisted of 11,304 individuals. The same individuals were re-interviewed but the sample was refreshed in wave 3 (2006/2007), wave 4 (2008/2009), and in wave 6 (2012/2013) to keep the original sample size in light of the attrition and death of some respondents. We use ELSA because it contains both mental and physical health variables. In addition, the population of individuals aged over 50 years is an ideal case study of the relationship between mental and physical health because of the relatively higher prevalence of physical and mental health conditions than amongst younger populations (World Health Organisation, 2013a, World Health Organisation, 2013b, Mental Health Foundation, 2009).

The measure of physical health is the six-item version ADL developed by Katz et al. (1963). The ADL index measures the difficulties in performing tasks required for personal self-care and independent living in every-day life. The functional assessment was based on individuals’ responses (yes/no) at each wave to the six item questions asking them for (1) difficulties in dressing, (2) walking across a room, (3) bathing or showering, (4) eating, (5) getting out of bed, and (6) using the toilet. The overall score (ADL) for each individual is calculated by summing across the item-specific responses (Zaninotti and Falaschetti, 2010). Thus the ADL index ranges from 0 (most difficulties and worst physical health) to 6 (least difficulties and best physical health).

The advantage of using ADL over SAH is that it measures physical activity only, whereas SAH might also reflect other factors. Pfarr et al. (2012) for instance find that reported SAH is, in addition to describing the physical health status as its primary goal, affected by health behaviour, health care utilisation and mental health status, and thus a function of both mental and physical health. Another advantage of using ADL is the steep increase in prevalence rates with advancing age (Rivlin et al., 1988).^1^

We measure mental health using a validated eight-item version of the CES-D scale developed by Radloff (1977).^2^ CES-D is a depression screening test collected in every wave of the ELSA. Individuals answered yes/no if over the past week they had had symptoms associated with depression, including (1) if they felt depressed much of the time, (2) they felt everything they did was an effort, (3) their sleep was restless, (4) they were unhappy, (5) they felt lonely, (6) they enjoyed life, (7) they felt sad, and (8) if they could not get going. Responses were summed (Demakakos et al., 2010) for each individual to compute a total CES-D scale, ranging from zero (high depression and worst mental health status) to eight (no depression and best mental health status). The eight-item version of the CES-D scale has been validated in older populations and displays strong psychometric properties (Zivin et al., 2010, Hamer et al., 2012, Irwin et al., 1999, Lyness et al., 1997). It also has comparable reliability and validity to the widely used 20-item CES-D scale (Turvey et al., 1999, Steffick, 2015). The CES-D scale has been found to have a strong correlation with and similar patterns to the General Health Questionnaire (GHQ) in comparison studies (Papassotiropoulos and Heun, 1999, Head et al., 2013)I It has been used in a wide set of longitudinal studies and is a stable measure of depression over time (Armenta et al., 2014).

Both physical health and mental health have been shown to have a strong positive correlation with health behaviour variables such as physical activity (Jeoung et al., 2013, Durstine et al., 2013, Contoyannis and Jones, 2004). Omitting physical activity from the estimation could cause an upward bias in the estimated coefficients on past physical and mental health if they are correlated. We therefore include a four level ordinary scale measure (1 = sedentary job and no PA activity, 2 = mild PA or job with standing, 3 = Moderate PA or job with PA, 4 = Vigorous PA or heavy manual job) of physical activity at baseline which is constructed to reflect both physical activity on the job and during leisure time, and intended to reflect preferences for physical activity.

The baseline value of a social interaction index is included to reflect preferences for social interactions such as informal care received by friends, children or other relatives. The social interaction index is constructed following Banks et al. (2010) by summing over the frequency of interactions with friends, family and children (3 = at least once a week; 2 = once or twice a month 1 = every few months, once or twice a year 0 = less than once a year or never), and a binary variables which takes the value of one if the individual is married or in a relationship and lives together with a partner. The maximum value is 10, indicating the strongest possible social interaction. The lowest value is 0, indicating the lowest social interaction applicable to an individual who has no friends, children or family.

We also consider the following demographic characteristics: gender, an ethnicity variable which takes a value of one if the respondent is from a white ethnic group, whether the individual has a higher educational qualification, whether the individual has retired from work, the number of household members and age. Amongst socioeconomic characteristics we consider whether the individual has private insurance coverage, whether the individual is unemployed, and the log of annual equivalised real household income deflated by the Consumer Price Index with 2005 as base year. We also control for the regional location of the respondent (North, South, East and Midlands, London). The choice of our covariates was based on the literature on dynamic health effects (see for instance: Hauck and Rice, 2004, Contoyannis et al., 2004, Jones et al., 2006, Contoyannis and Li, 2011).

### Summary statistics

Table 1 provides the descriptive statistics by survey wave for the full sample. The full sample size for all years is 11,203. About 54% of our respondents are female and 56% are retired, with a low rate of unemployment amongst the working-age respondents. The modal respondent is in good mental and physical health, is white, has higher education, lives together with another person at his/her household, is physically active, and has modest social interaction. A small proportion of the sample purchases supplementary private health insurance. The exponentiated logarithm of the equivalised disposable real household income of £13,095 is lower than the UK wide equivalent of £17,305 per capita (OECD, 2015) because the population under study is older than the UK wide population. Splitting the sample by waves shows the effect of the refreshing the sample in waves 3 and 4, indicated by a decrease in average age. The sample average remains stable for physical health between 2002 and 2012 and increases for mental health from 2002 to 2008. The increase in good mental health in 2012 reflects the refreshment of the sample in that wave.

**Table 1:** Descriptive statistics.

Variables	Definition	2004	2006	2008	2010	2012	All years
		*n* = 7,152	*n* = 6,306	*n* = 6,348	*n* = 7,693	*n* = 7,389	*n* = 11,203
ADL	0–6 scale; with 0 = worst physical health and 6 = best physical health	5.67 (0.88)	5.66 (0.89)	5.68 (0.83)	5.69 (0.85)	5.68 (0.88)	5.75 (0.73)
CES	0–8 scale; with 0 = worst mental health and 8 = best mental health	6.41 (2.02)	6.59 (1.90)	6.68 (1.85)	6.55 (1.91)	6.73 (1.83)	6.59 (1.91)
ADL (T-1)	Past ADL	5.71 (0.71)	5.68 (0.86)	5.70 (0.83)	5.72 (0.79)	5.72 (0.78)	5.71 (0.81)
CES (T-1)	Past CES	6.59 (1.87)	6.41 (2.02)	6.60 (1.91)	6.70 (1.85)	6.62 (1.87)	6.59 (1.90)
Male	1 if male, 0 if female	0.46	0.45	0.45	0.46	0.45	0.46
White	1 if white, 0 otherwise	0.98	0.98	0.98	0.98	0.97	0.98
Age	Age of the respondent	65.6 (9.67)	67.03 (9.60)	66.56 (9.92)	66.77 (9.22)	68.22 (9.00)	66.84 (9.51)
Higher Education	1 if higher education (university +), 0 otherwise	0.53	0.55	0.59	0.61	0.61	0.58
Household Size	Number of household members including respondent	1.97 (0.80)	1.93 (0.79)	1.98 (0.84)	1.97 (0.80)	1.95 (0.78)	1.96 (0.80)
Private Insurance	1 if private insurance, 0 otherwise	0.16	0.15	0.15	0.14	0.12	0.15
Retired	1 if retired from work, 0 otherwise	0.53	0.57	0.56	0.58	0.64	0.58
North	1 if from northern England, 0 otherwise	0.31	0.30	0.29	0.29	0.28	0.30
South	1 if from southern England, 0 otherwise	0.29	0.29	0.39	0.29	0.29	0.29
East and Midlands	1 if from East and/or Midlands, 0 otherwise	0.32	0.33	0.34	0.34	0.34	0.34
London	1 if from London, 0 otherwise	0.09	0.09	0.08	0.08	0.08	0.08
Income	Yearly log equivalised real household income in GBP	9.43 (0.67)	9.44 (0.67)	9.50 (0.69)	9.51 (0.65)	9.52 (0.65)	9.48 (0.67)
Unemployed	1 if unemployed, 0 otherwise	0.01	0.01	0.01	0.01	0.01	0.01
Physical activity (baseline)	1 = sedentary job and no PA activity, 2 = mild PA or job with standing, 3 = Moderate PA or job with PA, 4 = Vigorous PA or heavy manual job	3.17 (0.68)	3.18 (0.68)	3.22 (0.67)	3.22 (0.67)	3.23 (0.67)	3.21 (0.67)
Social interaction (baseline)	0 = lowest social interaction; 10 = highest social interaction	6.72 (2.13)	6.70 (2.12)	6.73 (2.11)	6.70 (2.12)	6.70 (2.09)	6.71 (2.12)

Note: Descriptive statistics are on the sample of the estimated models. n indicates the number of individuals. Variable means (standard deviations).

## Empirical strategy

We estimate the following dynamic models for physical and mental health, respectively:

(1) $${\mathrm{ADL}}_{i\text{,}T}=\beta_{0}+\beta_{1}{\mathrm{ADL}}_{i\text{,}T\text{-}1}+\beta_{2}{\mathrm{CES}}_{i\text{,}T\text{-}1}+X_{i\text{,}T}\beta_{3}+\beta_{4}{\mathrm{ADL}}_{i\text{,}T=0}+\beta_{5}{\mathrm{CES}}_{i\text{,}T=0}+\beta_{6}{\mathrm{PA}}_{i\text{,}T=0}+\beta_{7}{\mathrm{SI}}_{i\text{,}T=0}+v_{i\text{,}T}$$

(2) $${\mathrm{CES}}_{i\text{,}T}=\gamma_{0}+\gamma_{1}{\mathrm{ADL}}_{i\text{,}T\text{-}1}+\gamma_{2}{\mathrm{CES}}_{i\text{,}T\text{-}1}+X_{i\text{,}T}\gamma_{3}+\gamma_{4}{\mathrm{ADL}}_{i\text{,}T=0}+\gamma_{5}{\mathrm{CES}}_{i\text{,}T=0}+\gamma_{6}{\mathrm{PA}}_{i\text{,}T=0}+\gamma_{7}{\mathrm{SI}}_{i\text{,}T=0}+\rho_{i\text{,}T}$$

where ${\mathrm{ADL}}_{i\text{,}T\text{-}1}$ and ${\mathrm{CES}}_{i\text{,}T\text{-}1}$ are the one period lagged values, assuming that lagged health captures the state dependence in health which follows a first-ordered Markov process as commonly assumed in literature (see for example, Contoyannis et al., 2004). A lagged coefficient close to unity implies complete state dependence whereas a coefficient close to zero implies low state dependence. The vector Xcontains the control variables. ${\mathrm{ADL}}_{i\text{,}T=0}$ and ${\mathrm{CES}}_{i\text{,}T=0}$ are the initial conditions. Controlling for them conditions heterogeneity in health on the initial condition (Wooldridge, 2005). ${\mathrm{PA}}_{i\text{,}T=0}$captures investments in physical health at baseline, also addressing reverse causality with the explanatory variable. ${\mathrm{SI}}_{i\text{,}T=0}$, the social interaction index at baseline, captures the mental health investment of the individual. ${\mathrm{PA}}_{i\text{,}T=0}$ and ${\mathrm{SI}}_{i\text{,}T=0}$ condition the endogenous choice in health investments on the initial condition.

Adding ${\mathrm{CES}}_{i\text{,}T\text{-}1}$ to Eq. (1) and ${\mathrm{ADL}}_{i\text{,}T\text{-}1}$ to Eq. (2) measures the cross-effects of mental and physical health on each other. Adding these components could reduce the unobserved heterogeneity.

We use three models. First, we start with a pooled OLS which should lead to upwardly biased coefficients $\beta_{1}\text{,}\gamma_{1}$ and $\beta_{2}\text{,}\gamma_{2}$ because of the correlation of ${\mathrm{ADL}}_{i\text{,}T\text{-}1}$ and ${\mathrm{CES}}_{i\text{,}T\text{-}1}$ with the error term. This is the case if there is serial correlation caused by unobserved individual effects.

Second, to partially overcome the bias of individual unobserved heterogeneity, we assume random effects (RE) with the error term $v_{i\text{,}T}=\mu_{i}+\varepsilon_{i\text{,}T}\text{,}(\rho_{i\text{,}T}=\mu_{i}^{\prime }+\varepsilon_{i\text{,}T}^{\prime })\text{,}$ composed of the time-invariant individual component $\mu_{i}(\mu_{i}^{\prime })$and the time-varying error $\varepsilon_{i\text{,}T}(\varepsilon_{i\text{,}T}^{\prime })$. Decomposing the error variance gives the opportunity to understand how the cross-effects impact onthe individual specific unobserved heterogeneity in health.^3^ RE rules out any correlation of the variation across individuals with the independent variables. This assumes that the time-invariant individual component $\mu_{i}$ is not correlated with the regressors. The advantage of using RE for our model estimation is that important individual time-invariant attributes, in our case gender, education, the ethnicity dummy, can be included as explanatory variables in the estimation.^4^ Furthermore, RE give us the opportunity to better understand the differences between individuals’ mental and physical health stocks on present physical health as RE adds more weight to the between estimator of entities.

We report standardised coefficient values for our continuous variables in the RE estimation. Fully standardised coefficients for constant variables condition the estimated coefficient on a zero mean and a one unit standard deviation. Standardization is important in order to compare magnitudes across different variables of different scales.^5^ For instance, in the physical health equation, standardising allows us to compare the importance of the effect of including past mental health in comparison to other deterministic health variables such as health investments, age or income.

In a further analysis, we decompose the ADL and CES indices into their binary component variables and run linear probability models for each component. We use each of the six ADL components as separate dependent outcomes including the full set of eight lagged CES components as independent variables. We then use each of the eight CES binary-components as dependent variables and include each of the six lagged ADL components. This produces a matrix of coefficients and provides a richer description of the mental health-physical health relationship. For these models we use the full specification with random effects and the full set of covariates.

Third, we use RE ordered probit models. In using the ordered probit model, we examine potential non-linearities in state-dependence and in the relation between physical and mental health. We compute average partial effects (APEs) for each of the categories of ADL and CES-D. We report for ADL the levels low (0,1), medium (3), and high (5,6); similarly for CES-D (high (0,1), medium (4), and low (7,8)). For continuous variables, APEs are calculated by taking the derivative of the ordered probit probabilities with respect to the dependent variable. The APEs of discrete regressors are calculated by taking the differences of the derivatives between the groups.

For each of the three models we consider the following specifications. Firstly, we examine the relationships between past physical health and present physical health, and between past mental health and present mental health, controlling for the initial health conditions. Secondly, we add the set of covariates to mitigate individual unobserved heterogeneity. Finally, we include past mental health in the physical health equation and past physical health in mental health equation to estimate the cross-effects and their contributions to unobserved heterogeneity.^6^

## Results

### Linear models

The estimated coefficient on past physical health (Table 2) is always statistically significant and positively correlated with present physical health. The coefficient on past ADL in model (1) without covariates shows higher magnitude than the equivalent in (2) because of the upward bias in the OLS estimate. Adding covariates shows that past ADL picked up individual effects and hence caused upward bias in both models. About 22% of unobserved heterogeneity in physical health is accounted for by individual heterogeneity in the RE model. The coefficient of the past dependent variable is 0.513 in the OLS model and 0.369 in the RE model. The OLS estimate of the past dependent variable reflects both state dependence and individual heterogeneity.

**Table 2:** Linear regression models for physical health.

	Pooled (1)		RE (2)		RE (3)
	Without covariates	With covariates	Without covariates	With covariates	With lagged CES
ADL (T-1)	0.528⁎⁎⁎	0.513⁎⁎⁎	0.382⁎⁎⁎	0.369⁎⁎⁎	0.357⁎⁎⁎
	(0.013)	(0.013)	(0.015)	(0.015)	(0.015)
CES (T-1)					0.029⁎⁎⁎
					(0.003)
Male		0.004		0.005	−0.012
		(0.007)		(0.009)	(0.009)
White		−0.005		−0.007	−0.032
		(0.030)		(0.035)	(0.034)
Age		0.022⁎⁎⁎		0.028⁎⁎⁎	0.025⁎⁎⁎
		(0.006)		(0.007)	(0.006)
Age Squared		−0.000⁎⁎⁎		−0.000⁎⁎⁎	−0.000⁎⁎⁎
		(0.000)		(0.000)	(0.000)
Higher Education		0.031⁎⁎⁎		0.036⁎⁎⁎	0.024⁎⁎
		(0.008)		(0.010)	(0.009)
Household Size		0.003		0.005	−0.002
		(0.005)		(0.006)	(0.006)
Private Insurance		0.010		0.011	0.006
		(0.009)		(0.010)	(0.010)
Retired		0.021⁎⁎		0.023⁎⁎	0.019⁎
		(0.010)		(0.010)	(0.010)
Income		0.016⁎⁎⁎		0.018⁎⁎⁎	0.009
		(0.006)		(0.006)	(0.006)
Unemployed		−0.014		−0.007	0.006
		(0.031)		(0.031)	(0.031)
Social Interaction (baseline)		0.004⁎⁎		0.006⁎⁎	0.004
		(0.002)		(0.002)	(0.002)
Physcial Activity (baseline)		0.056⁎⁎⁎		0.068⁎⁎⁎	0.057⁎⁎⁎
		(0.006)		(0.007)	(0.007)
ADL (baseline)	0.212⁎⁎⁎	0.202⁎⁎⁎	0.309⁎⁎⁎	0.293⁎⁎⁎	0.280⁎⁎⁎
	(0.013)	(0.013)	(0.017)	(0.017)	(0.017)
CES (baseline)					0.014⁎⁎⁎
					(0.003)
Constant	1.445⁎⁎⁎	0.624⁎⁎⁎	1.703⁎⁎⁎	0.643⁎⁎⁎	0.838⁎⁎⁎
	(0.063)	(0.216)	(0.079)	(0.247)	(0.245)
Year	YES	YES	YES	YES	YES
Region		YES		YES	YES
Observations	34,885	34,885	34,885	34,885	34,885
Individuals	11,203	11,203	11,203	11,203	11,203
$\sigma_{\mu }$			0.225	0.224	0.22
$\sigma_{\varepsilon }$			0.55	0.548	0.548
ρ			0.144	0.143	0.139

Robust standard errors in parentheses.

⁎⁎⁎ *p* < 0.01.

⁎⁎ *p* < 0.05.

⁎ *p* < 0.1.

In both models current physical health is explained by higher education, the baseline level of social interaction, baseline physical activity, income, retirement, and age. Better health is associated with higher levels of physical activity and higher social interaction. The baseline value of physical health in column (4) has a relative large coefficient which indicates state-dependence in physical health driven by individual heterogeneity.

Adding the one period lagged value of mental health shows a positive correlation of past mental health with present physical health. This indicates that better mental health in the past is associated with a higher level of current physical health).^7^ Adding past mental health explains about 0.4% of the individual unobserved heterogeneity as ρ drops to 13.9% in the RE. Adding past mental health only marginally increases the explained variation in physical health.

The coefficient on past physical health is slightly smaller after including past mental health (Model (3) in Table 2). Thus, mental health only partly explains the association between past and present physical health. Adding past mental health does not change the signs of the estimated coefficients on the other covariates, but the coefficients on income and social interaction lose statistical significance and there is a marginal decrease in the magnitude of the coefficient on physical activity. The initial value of ADL is slightly reduced but remains larger than the lagged coefficient in explaining present health. For a standard deviation (SD) change in the stock of past mental health, present physical health changes by 0.08 SD, which is larger than the magnitude compared to the effect of baseline physical activity, for which a one SD change increases physical health by 0.05 SD. Comparing the fully standardised coefficient value to the coefficients on binary independent variables shows a similar picture. The magnitude of the standardised effect of past mental health on present physical health is more than three times the magnitude of the coefficient on higher education (0.024).

Table 3 presents the linear probability models for the six ADL components. Only the coefficients on the lagged components of the CES-D scale are presented. Having the feeling that everything takes an effort (CES effort T-1) in the past increases the probability to have physical functioning problems in all six ADL components at present. The probability of having problems with bathing, eating or going to bed at present is increased for individuals that did not enjoy life in the previous period (CES not enjoy life T-1). Being unhappy in the past (CES not happy T-1) is not significantly correlated with any ADL component. Feeling depressed in the past increases the probability of problems going to bed at present.

**Table 3:** Linear probability models for physical health components.

Dependent variable	Component of the CES scale							
	CES depressed (T-1)	CES effort (T-1)	CES restless (T-1)	CES not happy (T-1)	CES lonely (T-1)	CES not enjoy life (T-1)	CES sad (T-1)	CES not get going (T-1)
ADL shoe/sock	0.001	0.045⁎⁎⁎	0.024⁎⁎⁎	−0.007	0.006	0.012	0.001	0.025⁎⁎⁎
	(0.007)	(0.006)	(0.004)	(0.008)	(0.006)	(0.009)	(0.005)	(0.006)
ADL walking	0.002	0.012⁎⁎⁎	0.0002	0.002	−0.0001	0.005	−0.0036	0.005
	(0.004)	(0.0034)	(0.002)	(0.004)	(0.004)	(0.005)	(0.003)	(0.003)
ADL bath/shower	0.004	0.039⁎⁎⁎	0.012⁎⁎⁎	0.004	0.014⁎⁎	0.015⁎	−0.0025	0.012⁎⁎
	(0.004)	(0.006)	(0.006)	(0.007)	(0.006)	(0.008)	(0.005)	(0.005)
ADL eating	0.003	0.007⁎⁎	0.001	−0.002	−0.0001	0.011⁎⁎	−0.004⁎	0.001
	(0.003)	(0.003)	(0.003)	(0.002)	(0.004)	(0.004)	(0.002)	(0.003)
ADL bed	0.0002⁎⁎	0.019⁎⁎⁎	0.013⁎⁎⁎	−0.009	0.008	0.026⁎⁎⁎	0.001	0.013⁎⁎⁎
	(0.004)	(0.005)	(0.003)	(0.006)	(0.005)	(0.007)	(0.004)	(0.004)
ADL toilet	−0.0001	0.014⁎⁎⁎	0.002	0.0017	0.005	0.007	−0.003	0.002
	(0.004)	(0.004)	(0.002)	(0.005)	(0.004)	(0.005)	(0.003)	(0.003)

Robust standard errors in parentheses.

Each row represents a separate regression model. Models also contain the lagged value of ADL, and the covariates: Male, white, age, age squared, higher education, household size, private insurance, retired, income, unemployed, social interaction at baseline, physical activity at baseline, CES at baseline and ADL at baseline.

⁎⁎⁎ *p* < 0.01.

⁎⁎ *p* < 0.05.

⁎ *p* < 0.1.

The estimated coefficient on past mental health (Table 4) is always statistically significant and positively correlated with present mental health. We observe similar patterns as in the physical health estimation for the OLS, adding additional covariates and switching to the RE models. About 45% of unobserved heterogeneity is accounted for by individual heterogeneity in the RE model. In both models, all of the covariates are statistically significant. Better mental health is also associated with higher levels of physical activity and social interaction at baseline. The baseline value of mental health has a larger coefficient than that on the previous period’s mental health.

**Table 4:** Linear regression models for mental health.

	Pooled (1)		RE (2)		RE (3)
	Without covariates	With covariates	Without covariates	With covariates	With lagged ADL
ADL (T-1)					0.182⁎⁎⁎
					(0.017)
CES (T-1)	0.377⁎⁎⁎	0.358⁎⁎⁎	0.256⁎⁎⁎	0.245⁎⁎⁎	0.236⁎⁎⁎
	(0.008)	(0.008)	(0.008)	(0.008)	(0.008)
Male		0.149⁎⁎⁎		0.162⁎⁎⁎	0.174⁎⁎⁎
		(0.017)		(0.021)	(0.021)
White		0.115∗		0.132⁎	0.141⁎
		(0.069)		(0.078)	(0.078)
Age		0.044⁎⁎⁎		0.055⁎⁎⁎	0.054⁎⁎⁎
		(0.013)		(0.014)	(0.014)
Age Squared		−0.000⁎⁎⁎		−0.000⁎⁎⁎	−0.000⁎⁎⁎
		(0.000)		(0.000)	(0.000)
Higher Education		0.089⁎⁎⁎		0.101⁎⁎⁎	0.088⁎⁎⁎
		(0.018)		(0.022)	(0.022)
HHsize		0.087⁎⁎⁎		0.102⁎⁎⁎	0.102⁎⁎⁎
		(0.012)		(0.014)	(0.014)
Private Insurance		0.062⁎⁎⁎		0.069⁎⁎⁎	0.061⁎⁎
		(0.023)		(0.024)	(0.024)
Retired		0.098⁎⁎⁎		0.104⁎⁎⁎	0.099⁎⁎⁎
		(0.022)		(0.024)	(0.024)
Income		0.122⁎⁎⁎		0.121⁎⁎⁎	0.120⁎⁎⁎
		(0.015)		(0.015)	(0.015)
Unemployed		−0.126		−0.132	−0.163
		(0.115)		(0.115)	(0.115)
Social Interaction (baseline)		0.012⁎⁎⁎		0.014⁎⁎⁎	0.015⁎⁎⁎
		(0.004)		(0.005)	(0.005)
Physical Activity (baseline)		0.109⁎⁎⁎		0.125⁎⁎⁎	0.078⁎⁎⁎
		(0.013)		(0.016)	(0.016)
CES (baseline)	0.253⁎⁎⁎	0.234⁎⁎⁎	0.333⁎⁎⁎	0.305⁎⁎⁎	0.286⁎⁎⁎
	(0.008)	(0.008)	(0.009)	(0.009)	(0.009)
ADL (baseline)					0.067⁎⁎⁎
					(0.020)
Constant	2.499⁎⁎⁎	−0.615	2.803⁎⁎⁎	−0.794	−1.900⁎⁎⁎
	(0.049)	(0.461)	(0.061)	(0.506)	(0.505)
Year	YES	YES	YES	YES	YES
Region		YES		YES	YES
Observations	34,885	34,885	34,885	34,889	34,888
Individuals	11,203	11,203	11,203	11,203	11,203
$\sigma_{\mu }$			0.47	0.45	0.43
$\sigma_{\varepsilon }$			1.3	1.29	1.29
ρ			0.12	0.11	0.1

Robust standard errors in parentheses.

⁎⁎⁎ *p* < 0.01.

⁎⁎ *p* < 0.05.

⁎ *p* < 0.1.

Focussing on model (3), the past value of physical health shows a relatively large and positive correlation with present mental health. Adding the physical health component to the mental health equation explains 2% of unobserved heterogeneity (ρ drops from 11% to 10%). Hence the explained variation in mental health increases marginally. Lagged physical health shows a strong association with present mental health. Amongst the three health variables, the baseline value of mental health has the strongest relationship (0.286) with present mental health. Similar to the linear estimation of physical health, adding the stock of past physical health to the mental health equation shows strong magnitude effects compared to the other estimators. A SD change in the stock of past physical health produces a 0.08 SD change in present mental health, which is twice the fully standardised effects of income (0.04) and physical activity (0.04), and four times the effect of baseline social interaction (0.02) with mental health. Compared to the binary outcomes, the magnitude of the standardised effect of past physical health on mental health has a larger magnitude than holding a private insurance (0.06) and a similar magnitude as being higher educated (0.08).

Table 5 presents the linear probability models for the eight components of the CES scale. Only the coefficients on the lagged components of the ADL scale are presented. Past problems using shoes or socks (ADL shoe/sock T-1) have a significant positive effect on the probability of all CES components. Problems with walking in the past (AFL walking T-1) significantly increases the probability of feeling everything is an effort, feeling that one does not get going and that life is not enjoyable at present. Problems taking a shower or having a bath in the past (ADL bath/shower T-1) increase the probability to feel depressed, that everything is an effort, of being restless, feeling lonely, of not enjoying life, and of not getting going at present. Problems going to bed in the past (ADL bed T-1) are positively correlated with the probability to feel depressed, to perceive that everything takes an effort, to feel restless, to feel sad, and to feel of not getting going. Neither problems of using the toilet (ADL toilet T-1) nor eating problems (ADL eating T-1) have significant effects on any of the CES components.

**Table 5:** Linear probability models for mental health components.

Dependent variable	Components of the ADL scale					
	ADL shoe/sock (T-1)	ADL walking (T-1)	ADL bath/shower (T-1)	ADL eating (T-1)	ADL bed (T-1)	ADL toilet (T-1)
CES depressed	0.041⁎⁎⁎	−0.014	0.034⁎⁎⁎	0.006	0.036⁎⁎⁎	0.001
	(0.01)	(0.02)	(0.01)	(0.023)	(0.013)	(0.02)
CES effort	0.091⁎⁎⁎	0.064⁎⁎⁎	0.07⁎⁎⁎	0.035	0.082⁎⁎⁎	0.018
	(0.01)	(0.022)	(0.012)	(0.024)	(0.014)	(0.019)
CES restless	0.077⁎⁎⁎	0.01	0.032⁎⁎⁎	−0.028	0.1⁎⁎⁎	−0.021
	(0.01)	(0.022)	(0.01)	(0.024)	(0.014)	(0.018)
CES not happy	0.029⁎⁎⁎	0.011	0.004	−0.031⁎	0.0001	0.004
	(0.01)	(0.02)	(0.01)	(0.02)	(0.011)	(0.014)
CES lonely	0.021⁎⁎⁎	0.006	0.017⁎	0.001	0.006	0.002
	(0.007)	(0.019)	(0.009)	(0.021)	(0.011)	(0.015)
CES not enjoy life	0.023⁎⁎⁎	0.042⁎⁎	0.033⁎⁎⁎	−0.006	0.005	0.008
	(0.007)	(0.019)	(0.009)	(0.02)	(0.011)	(0.015)
CES sad	0.04⁎⁎⁎	0.015	0.009	0.028	0.023⁎	0.011
	(0.009)	(0.021)	(0.01)	(0.024)	(0.013)	(0.018)
CES not get going	0.082⁎⁎⁎	0.085⁎⁎⁎	0.086⁎⁎⁎	−0.003	0.068⁎⁎⁎	−0.002
	(0.01)	(0.023)	(0.011)	(0.024)	(0.015)	(0.019)

Robust standard errors in parentheses.

Each row represents a separate regression model. Models also contain the lagged value of CES, and the covariates: Male, white, age, age squared, higher education, household size, private insurance, retired, income, unemployed, social interaction at baseline, physical activity at baseline, CES at baseline and ADL at baseline.

⁎⁎⁎ *p* < 0.01.

⁎⁎ *p* < 0.05.

⁎ *p* < 0.1.

### Non-linear models

In the non-linear model (Table 6), ADL and CES-D are increasing in their past values (Models (1) and (3)). After adding covariates, the magnitude of the coefficients of past ADL level-coefficients in model (1) and past CES coefficients in model (3) increase.

**Table 6:** Non-linear random effects estimation of physical and mental health.

	RE Oprobit ADL(1)		RE Oprobit ADL(2)	RE Oprobit CES (3)		RE Oprobit CES(4)
	Without cov.	With cov.	With CES (T-1)	Without cov.	With cov.	With ADL (T-1)
ADL(T-1) = 1	−0.09	−0.035	0.0016			−0.145
	(0.187)	(0.186)	(0.184)			(0.175)
ADL(T-1) = 2	−0.003	0.102	0.155			−0.154
	(0.178)	(0.177)	(0.175)			(0.165)
ADL(T-1) = 3	0.157	0.302⁎	0.348⁎⁎			−0.047
	(0.175)	(0.174)	(0.172)			(0.162)
ADL(T-1) = 4	0.198	0.363⁎⁎	0.405⁎⁎			−0.041
	(0.174)	(0.173)	(0.172)			(0.16)
ADL(T-1) = 5	.459⁎⁎⁎	0.618⁎⁎⁎	0.63⁎⁎⁎			−0.004
	(0.175)	(0.174)	(0.173)			(0.159)
ADL(T-1) = 6	1.07⁎⁎⁎	1.146⁎⁎⁎	1.139⁎⁎⁎			0.25
	(0.181)	(0.179)	(0.178)			(0.159)
CES(T-1) = 1			0.08⁎⁎⁎	0.083	0.104	0.096
			(0.087)	(0.07)	(0.071)	(0.07)
CES(T-1) = 2			0.107⁎⁎⁎	0.11	0.113⁎	0.109⁎
			(0.084)	(0.068)	(0.068)	(0.068)
CES(T-1) = 3			0.097⁎⁎⁎	0.11⁎	0.131⁎	0.131∗
			(0.082)	(0.067)	(0.067)	(0.067)
CES(T-1) = 4			0.159⁎⁎⁎	0.174⁎⁎⁎	0.191⁎⁎⁎	0.187⁎⁎⁎
			(0.081)	(0.066)	(0.066)	(0.065)
CES(T-1) = 5			0.222⁎⁎⁎	0.251⁎⁎⁎	0.271⁎⁎⁎	0.278⁎⁎⁎
			(0.079)	(0.065)	(0.064)	(0.064)
CES(T-1) = 6			0.254⁎⁎⁎	0.3⁎⁎⁎	0.315⁎⁎⁎	0.31⁎⁎⁎
			(0.078)	(0.064)	(0.064)	(0.064)
CES(T-1) = 7			0.371⁎⁎⁎	0.407⁎⁎⁎	0.408⁎⁎⁎	0.398⁎⁎⁎
			(0.078)	(0.065)	(0.065)	(0.065)
CES(T-1) = 8			0.543⁎⁎⁎	0.649⁎⁎⁎	0.64⁎⁎⁎	0.633⁎⁎⁎
			(0.079)	(0.068)	(0.068)	(0.068)
Year	YES	YES	YES	YES	YES	YES
Region		YES	YES		YES	YES
Observations	34,888	34,888	34,888	34,888	34,888	34,888
Individuals	11,203	11,204	11,204	11,204	11,204	11,204
cut1	−0.237	0.96	0.146	−0.1⁎⁎⁎	2.502⁎⁎⁎	2.79⁎⁎⁎
	(0.18)	(0.645)	(0.64)	(0.066)	(0.429)	(0.451)
cut2	0.536⁎⁎	1.72⁎⁎⁎	0.9	−0.368⁎⁎⁎	3.129⁎⁎⁎	3.412⁎⁎⁎
	(0.176)	(0.645)	(0.64)	(0.064)	(0.429)	(0.451)
cut3	1.085⁎⁎⁎	2.268⁎⁎⁎	1.444⁎⁎	0.066	3.563⁎⁎⁎	3.852⁎⁎⁎
	(0.176)	(0.645)	(0.64)	(0.063)	(0.429)	(0.451)
cut4	1.691⁎⁎⁎	2.873⁎⁎⁎	2.05⁎⁎⁎	0.422⁎⁎⁎	3.92⁎⁎⁎	4.208⁎⁎⁎
	(0.176)	(0.646)	(0.64)	(0.063)	(0.428)	(0.452)
cut5	2.336⁎⁎⁎	3.521⁎⁎⁎	2.70⁎⁎⁎	0.775⁎⁎⁎	4.274⁎⁎⁎	4.562⁎⁎⁎
	(0.177)	(0.647)	(0.64)	(0.064)	(0.430)	(0.452)
cut6	3.202⁎⁎⁎	4.405⁎⁎⁎	3.58⁎⁎	1.21⁎⁎⁎	4.72⁎⁎⁎	5.0⁎⁎
	(0.179)	(0.647)	(0.64)	(0.064)	(0.430)	(0.452)
cut7				1.69⁎⁎⁎	5.196	5.48⁎
				(0.064)	(0.430)	(0.452)
cut8				2.565⁎⁎⁎	6.073	6.359
				(0.064)	(0.430)	(0.453)
Log LH	−18,704.35	−18,274.83	−18,079.541	−49,522.65	−49,194.748	−49,046.694

Standard errors in parentheses.

⁎⁎⁎ *p* < 0.01.

⁎⁎ *p* < 0.05.

⁎ *p* < 0.1.

The size and statistical significance of the regressors in the nonlinear models are similar to the linear models. There is a U-shaped relationship between ADL and age as its squared value is significantly and positively correlated with present physical health with turning point at age 90. The U-shaped relationship is also observed in model (3) for CES and age, with a turning point at age 71.

Adding the dynamic mental health component in model (2) does not change the order of the past ADL-level-coefficients, and increase the magnitude of the ADL-coefficients. The ${\mathrm{CES}}_{T\text{-}1}$ dummies are statistically significant from moderate past mental health on (${\mathrm{CES}}_{T\text{-}1}=4)$ and show the expected association with the present physical health. Compared to the worst possible mental health status (${\mathrm{CES}}_{T\text{-}1}=0$), the effect of better mental health on present physical is positive and increasing in its magnitude.

Adding the dynamic physical health component in model (4) decreases the magnitude of the coefficients on past mental health. Better past mental health with worst past mental health at baseline is associated with better present mental health. None of the ADL-coefficients is significant.^8^

We report APEs for low, medium and high levels of ADL (Table 7) and similarly for CES (Table 8). In Table 7, both past physical and mental health have a strong effect on present physical health. For past physical health, the effect shows significance from a moderate level of health upwards. We observe for past mental health significant average partial effects over all levels of past mental health. The estimated APEs on the lags of ADL shift the distribution of current physical health to the right, indicating better physical health. Moderate past physical health (*ADL_T_*_−1_ = 4) decreases the probability of worst present physical health by 0.002 compared to an average respondent with worst past physical health. The probability decreases by 0.003 for best past physical health. For best present physical health, moderate past physical health increases the probability to have best possible present physical by 0.113 and for best past physical health the probability increases by 0.252.

**Table 7:** Average Partial Effects of past levels of mental and past physical health on levels of current physical health.

	ADL = 0	ADL = 1	ADL = 3	ADL = 5	ADL = 6
ADL(T-1) = 1	−0.000	−0.000	−0.000	−0.000	0.000
	(0.001)	(0.002)	(0.0083)	(0.026)	(0.057)
ADL(T-1) = 2	−0.001	−0.0017	−0.0064⁎	−0.022	0.046
	(0.001)	(0.002)	(0.0079)	(0.024)	(0.054)
ADL(T-1) = 3	−0.002	−0.0033	−0.0129⁎	−0.049⁎⁎	0.099⁎
	(0.001)	(0.002)	(0.008)	(0.024)	(0.0532)
ADL(T-1) = 4	−0.002⁎	−0.0037⁎	−0.0146⁎⁎	−0.057⁎⁎	0.113⁎⁎
	(0.001)	(0.002)	(0.008)	(0.024)	(0.053)
ADL(T-1) = 5	−0.0023⁎⁎	−0.005⁎⁎	−0.02⁎⁎	−0.087⁎⁎⁎	0.165⁎⁎⁎
	(0.001)	(0.002)	(0.0082)	(0.024)	(0.054)
ADL(T-1) = 6	−0.003⁎⁎⁎	−0.0068⁎⁎⁎	−0.028⁎⁎⁎	−0.142⁎⁎⁎	0.252⁎⁎⁎
	(0.001)	(0.002)	(0.008)	(0.025)	(0.056)
CES(T-1) = 1	−0.0003	−0.0005	−0.0015	−0.009	0.015
	(0.0003)	(0.0006)	(0.002	(0.009)	(0.017)
CES(T-1) = 2	−0.0004	−0.0007	−0.002	−0.012	0.02
	(0.0003)	(0.0005)	(0.002)	(0.009)	(0.016)
CES(T-1) = 3	−0.0004	−0.0006	−0.002	−0.011	0.018
	(0.0003)	(0.0005)	(0.002)	(0.0093)	(0.016)
CES(T-1) = 4	−0.001⁎	−0.001⁎⁎	−0.0028⁎	−0.017⁎	0.029⁎
	(0.0003)	(0.0005)	(0.002)	(0.009)	(0.016)
CES(T-1) = 5	−0.001⁎⁎	−0.0013⁎⁎	−0.0038⁎⁎	−0.024⁎⁎⁎	0.04⁎⁎⁎
	(0.0003)	(0.0005)	(0.002)	(0.009)	(0.015)
CES(T-1) = 6	−0.001⁎⁎⁎	−0.0015⁎⁎⁎	−0.0043⁎⁎⁎	−0.027⁎⁎⁎	0.045⁎⁎⁎
	(0.0003)	(0.0005)	(0.002)	(0.009)	(0.015)
CES(T-1) = 7	−0.0012⁎⁎⁎	−0.002⁎⁎⁎	−0.0059⁎⁎⁎	−0.037⁎⁎⁎	0.063⁎⁎⁎
	(0.0003)	(0.0005)	(0.002)	(0.009)	(0.015)
CES(T-1) = 8	−0.0015⁎⁎⁎	−0.003⁎⁎⁎	−0.008⁎⁎⁎	−0.051⁎⁎⁎	0.085⁎⁎⁎
	(0.0003)	(0.0005)	(0.0015)	(0.009)	(0.015)

Robust standard errors in parentheses.

⁎⁎⁎ *p* < 0.01.

⁎⁎ *p* < 0.05.

⁎ *p* < 0.1.

**Table 8:** Average Partial Effects of past levels mental and past physical health on levels of current mental health.

	CES = 0	CES = 1	CES = 4	CES = 7	CES = 8
ADL(T-1) = 1	0.003	0.0042	0.0069	0.0025	−0.046
	(0.003)	(0.0048)	(0.008)	(0.0049)	(0.056)
ADL(T-1) = 2	0.003	0.0045	0.0073	0.0025	−0.049
	(0.003)	(0.004)	(0.0076)	(0.0049)	(0.053)
ADL(T-1) = 3	0.0008	0.0013	0.0022	0.0012	−0.015
	(0.003)	(0.004)	(0.0074)	(0.0049)	(0.052)
ADL(T-1) = 4	0.0007	0.001	0.0019	0.0011	−0.013
	(0.003)	(0.004)	(0.0073)	(0.0049)	(0.051)
ADL(T-1) = 5	0.0001	0.0001	0.0002	0.0002	−0.002
	(0.051)	(0.004)	(0.0073)	(0.0049)	(0.051)
ADL(T-1) = 6	0.081	−0.006	−0.011	−0.013⁎⁎⁎	0.081
	(0.051)	(0.004)	(0.0073)	(0.0049)	(0.051)
CES(T-1) = 1	−0.002	−0.003	−0.005	0.0014	0.03
	(0.0014)	(0.0024)	(0.004)	(0.0016)	(0.022)
CES(T-1) = 2	−0.0021	−0.0034	−0.006	0.0014	0.034⁎
	(0.0014)	(0.0022)	(0.0037)	(0.002)	(0.021)
CES(T-1) = 3	−0.0025⁎	−0.004⁎	−0.007⁎	0.0014	0.041⁎⁎
	(0.0014)	(0.0022)	(0.0037)	(0.002)	(0.021)
CES(T-1) = 4	−0.0034⁎⁎	−0.006⁎⁎⁎	−0.01⁎⁎⁎	0.001	0.059⁎⁎⁎
	(0.0013)	(0.0021)	(0.0036)	(0.002)	(0.02)
CES(T-1) = 5	−0.0047⁎⁎⁎	−0.008⁎⁎⁎	−0.015⁎⁎⁎	-0.002	0.089⁎⁎⁎
	(0.0013)	(0.0021)	(0.0036)	(0.0022)	(0.02)
CES(T-1) = 6	−0.0051⁎⁎⁎	−0.009⁎⁎⁎	−0.016⁎⁎⁎	−0.0027	0.1⁎⁎⁎
	(0.0013)	(0.0021)	(0.0036)	(0.0021)	(0.02)
CES(T-1) = 7	−0.0061⁎⁎⁎	−0.011⁎⁎⁎	−0.02⁎⁎⁎	−0.007⁎⁎⁎	0.13⁎⁎⁎
	(0.0013)	(0.0022)	(0.0037)	(0.002)	(0.02)
CES(T-1) = 8	−0.0081⁎⁎⁎	−0.015⁎⁎⁎	0.03⁎⁎⁎	−0.025⁎⁎⁎	0.21⁎⁎⁎
	(0.0014)	(0.0022)	(0.0039)	(0.002)	(0.02)

Robust standard errors in parentheses.

⁎⁎⁎ *p* < 0.01.

⁎⁎ *p* < 0.05.

⁎ *p* < 0.1.

Past mental health is also characterised by a rightward shift in the probabilities. Moderate past mental health (${\mathrm{CES}}_{T\text{-}1}=4$) decreases the probability to have worst present physical health by 0.001 compared to the base category of worst past mental health. For best past mental health the probability for worst physical health at present decreases by 0.0015. Looking at the best present health level, the probability increases by 0.029 for moderate past mental health, and for best past mental health the probability for best possible present health increases by 0.085.

In Table 8, past mental health has in most levels from poor-moderate mental health (${\mathrm{CES}}_{T\text{-}1}=3$) onwards a strong significant effect on current mental health with a shift of the distribution of current mental health to the right, indicating better mental health. Past physical health shows only a significant non-linear level impact on very good present mental health for best possible past physical health by reducing the probability by 0.013 compared to worst past ADL. Poor-moderate past mental health decreases the probability to have worst present mental health by 0.0025 compared to the baseline which is worst past mental health. For best past mental health the probability for worst mental health at present decreases by 0.0081. Looking at the best present mental health level, the probability increases by 0.0041 for poor-moderate past mental health, and for best past mental health the probability for best present health increases by 0.21 in comparison with worst past mental health.

## Conclusion

This paper has examined the interlinked dynamics of mental and physical health using a large longitudinal sample of the older population in England (ELSA, 2002–2013). Similar to Contoyannis et al. (2004), we find that 22% of the unexplained variation in physical health is explained by permanent individual unobserved heterogeneity. In mental health it is 43%. Mental health is an important determinant of physical health with stronger effects on physical health than other health deterministic variables. However, it only explains 0.4% of the unobserved heterogeneity in physical health. We find stronger effects of past physical health on present mental health. The magnitude of this effect is also stronger than of health investment variables or education with mental health. Addressing this cross-relationship reduces the unobserved heterogeneity in mental health by 2%.

Health dynamics significantly explain physical health and mental health. Physical health and mental health display modest state dependence in their respective past stocks. There are non-linear dynamic effects of both mental and physical health on physical health. For mental health, we observe non-linear dynamic effects in past mental health. Baseline levels of health have large effects, which provides further evidence of state-dependence in health and the importance of unmeasured individual characteristics such as genetic endowments.

We find moderate state dependence in physical health with the coefficient of past physical health being 0.357 and the coefficient on the baseline value of physical health being 0.28. The opposite holds for past mental health which has a coefficient closer to zero (0.029). This implies a weaker dependence of physical health on past mental health. The findings for dynamics in physical health concur with Contoyannis et al. (2004) who also find state dependence in physical health, though with a higher magnitude and stronger persistence. Including the stock of past mental health has important economic implications for explaining physical health which is shown by the stronger magnitude effects than physical activity or education when fully standardising. Linear probability model estimation for each of the components of ADL and CES (T-1) shows that the relationship is strongly driven by the feeling that everything takes an effort, and also by restlessness, not enjoying life and not getting on going.

The analysis of the effects of past mental health and past physical health on present mental health shows that mental health is modestly state dependent in both stocks. The coefficient of the past mental health component takes the value 0.236, indicating a modest state dependence of mental health on the outcome. The effect of baseline mental health on present mental health is substantially larger. This indicates a modest state-dependent relationship of past mental health with present mental health. A similar interpretation holds for the cross-effect with past physical health which is 0.181. The findings for dynamics in mental health stand in line with Hauck and Rice (2004) who also find state dependency of mental health of a similar magnitude. Including the stock of past physical health in the mental health equation shows strong(er) magnitude effects compared to the other regressors. Past physical health has stronger associations with present mental health than income or health investments. Decomposing the ADL index and regressing each of the present CES components on the lagged components of the ADL scale shows that having difficulties in dressing or in the every-day bathroom routine in the past contributes strongest to the probability of developing mental health problems at present.

A limitation of the paper is that the ELSA only collects information on the population aged 50 years and over limiting any conclusion to the older English population. Another limitation arises regarding the unobserved individual heterogeneity in health. Conclusions of the paper are therefore based on associations. We considered several methodologies addressing individual unobserved heterogeneity. Methodologies such as fixed effect models, and correlated random effects are not applicable because they are biased for estimating dynamic models when there is a small number of waves. Arellano-Bond estimation (Arellano and Bond, 1991) is not applicable either because of the small number of waves.

The moderate persistence of physical health supports the role for short run health interventions as these would be expected to have positive continuing effects on health. The non-linear effects in physical health show weaker effects at lower levels of present physical health from across all levels of past physical health. On the contrary, at higher present physical health the findings suggest stronger associations with better past physical health levels. This implies better health carries over to the following period whereas for worse physical health weaker effects are found. This then implies that at poor health levels continuing health interventions are necessary. This implies that policies focussing on the lower end of the distribution might have strongest effects.

The weak state dependence in the dynamic relation of stock of mental health with physical health suggests that policy interventions targeted on this relationship should rather look into continuous interventions as previously found by Golberstein and Busch (2014). Ongoing social interaction, especially in older age, hence shows not only importance for mental health outcomes but also for physical health because of the cross-effect on mental health. For the moderate persistence of mental health in both dynamic health stocks, health policies aiming at improving mental health might consider short-run interventions in both dimensions. The non-linear effects in mental health indicate that policies focussing on poor states of mental health can produce stronger positive marginal effects than at better levels of mental health.

The cross-effects between physical and mental health suggest that health policies that target either dimension might generate positive spill-overs in the other dimension. This is especially important for physical health interventions targeted at the older population because of their strong indirect effects on mental health outcomes.

Future studies should look into whether the dynamic dependencies between mental and physical health vary across different population groups, and whether improvements in physical or mental health generated by interventions have spillover effects.

## Footnotes

1 ADL has been criticised as being invariant to severity of physical health symptoms (White et al., 2011). However, we find that for this sample ADL is strongly correlated with more “objective” measures of physical health such as blood pressure, self-rated health and long standing conditions. We do not report these results here but they are available from the authors on request. Our results support Dennis et al. (2012) who find strong interrelation with functional health and systematic inflammation and muscle function representing physical health. ADL has been show to be a robust measure for physical health. Analysing the predictive power of different health measures on mortality, Cesari et al. (2008) find that ADL shows similar effects as self-rated health for the older population. Similarly, Dave et al. (2006) find that ADL is consistent with other physical health outcomes in the physical health – retirement relationship.

2 We tested the association with other depression scales such as the GHQ and found a strong positive correlation between these scales. CES-D is also a robust mental health measure. Galper et al. (2006) find similar results as ours by using CES-D and well-being as outcome measures to analyse the relationship of mental health with physical activity. Likewise do Dave et al. (2006) using different proxies for mental health, including CES-D, when analysing the effect of retirement on mental health outcomes.

3 $\rho =\frac{\sigma_{\mu }^{2}}{\sigma_{\mu }^{2}+\sigma_{∊}^{2}}$ measures the correlation of CES and ADL respectively over time. The closer the value is to zero the stronger is the contribution of permanent unobserved heterogeneity. The closer to zero the more that time-varying factors matter.

4 We did robustness tests using fixed effects, first-differentiating, and correlated random effects controlling for initial conditions and correlation in exogenous variables to remove the time-invariant unobserved heterogeneity. However, this leads to downward biased estimates of the lagged dependent variable for the low number of time *t* observations (Nickell, 1981, Angrist and Pischke, 2009, Contoyannis and Li, 2011).

5 To fully standardise the following transformation is applied: $\beta_{k}^{\prime }=\beta_{k}*\frac{{\text{SD}}_{\text{x}(\text{k})}}{{\text{SD}}_{\text{y}}}$.

6 We test for attrition effects by estimating balanced and unbalanced panels models, and by adding an attrition dummy to the equation. We did not find any qualitative change of the main coefficients in either specification.

7 Current physical health is likely to be influenced by the interaction of past physical with past mental health. We do not find a statistically significant effect of this interaction coefficient on current physical health.

8 A test of the joint significance of the ADL level-coefficients strongly rejects the null that the regressors are not jointly significant. This stands in line with the finding in the linear model that ADL (T-1) has a significant positive effect on present mental health.
